# Assembly of an atypical α-macroglobulin complex from *Pseudomonas aeruginosa*

**DOI:** 10.1038/s41598-017-18083-6

**Published:** 2018-01-11

**Authors:** Samira Zouhir, Mylène Robert-Genthon, Daniel Maragno Trindade, Viviana Job, Marko Nedeljković, Cécile Breyton, Christine Ebel, Ina Attrée, Andréa Dessen

**Affiliations:** 1 Brazilian Biosciences National Laboratory (LNBio), CNPEM, Campinas, São Paulo, Brazil; 2 University Grenoble Alpes, Bacterial Pathogenesis and Cellular Responses Group, Institut de Biosciences et Biotechnologies de Grenoble (BIG), Grenoble, France; 3 University Grenoble Alpes, CNRS, CEA, Institut de Biologie Structurale (IBS), F-38000 Grenoble, France

## Abstract

Alpha-2-macroglobulins (A2Ms) are large spectrum protease inhibitors that are major components of the eukaryotic immune system. Pathogenic and colonizing bacteria, such as the opportunistic pathogen *Pseudomonas aeruginosa*, also carry structural homologs of eukaryotic A2Ms. Two types of bacterial A2Ms have been identified: Type I, much like the eukaryotic form, displays a conserved thioester that is essential for protease targeting, and Type II, which lacks the thioester and to date has been poorly studied despite its ubiquitous presence in Gram-negatives. Here we show that MagD, the Type II A2M from *P. aeruginosa* that is expressed within the six-gene *mag* operon, specifically traps a target protease despite the absence of the thioester motif, comforting its role in protease inhibition. In addition, analytical ultracentrifugation and small angle scattering show that MagD forms higher order complexes with proteins expressed in the same operon (MagA, MagB, and MagF), with MagB playing the key stabilization role. A *P. aeruginosa* strain lacking *magB* cannot stably maintain MagD in the bacterial periplasm, engendering complex disruption. This suggests a regulated mechanism of Mag complex formation and stabilization that is potentially common to numerous Gram-negative organisms, and that plays a role in periplasm protection from proteases during infection or colonization.

## Introduction

Alpha-2-macroglobulins (A2Ms) are broad-spectrum protease inhibitors present in all metazoans ranging from insects to humans, and play key roles in host defense^1^. A2Ms are essential for trapping proteases secreted by invading microorganisms, thwarting the infectious process^2^. Eukaryotic A2Ms also recognize ‘self’ proteases, thus regulating inflammatory and blood clotting events. Their ubiquitous action derives from the fact that they are characterized by a highly reactive thioester bond (the CXEQ motif), as well as a bait region whose sequence is recognizable by a large number of proteases^3^.

Upon recognition and cleavage of the bait region by an attacking protease, the thioester bond becomes exposed and subsequently cross-linked to the enzyme, causing the A2M to trap the attacking protease in a cage-like structure^4,5^. This mechanism ensures that proteases secreted by infecting microorganisms are cleared effectively, and is thought to be part of an innate immune system that predates the immunoglobulin-based system^3^. A2Ms are members of a superfamily of proteins involved in other defense mechanisms, such as the complement cascade (i.e., C3/C5 convertases^3,6^).

**Figure 1:**
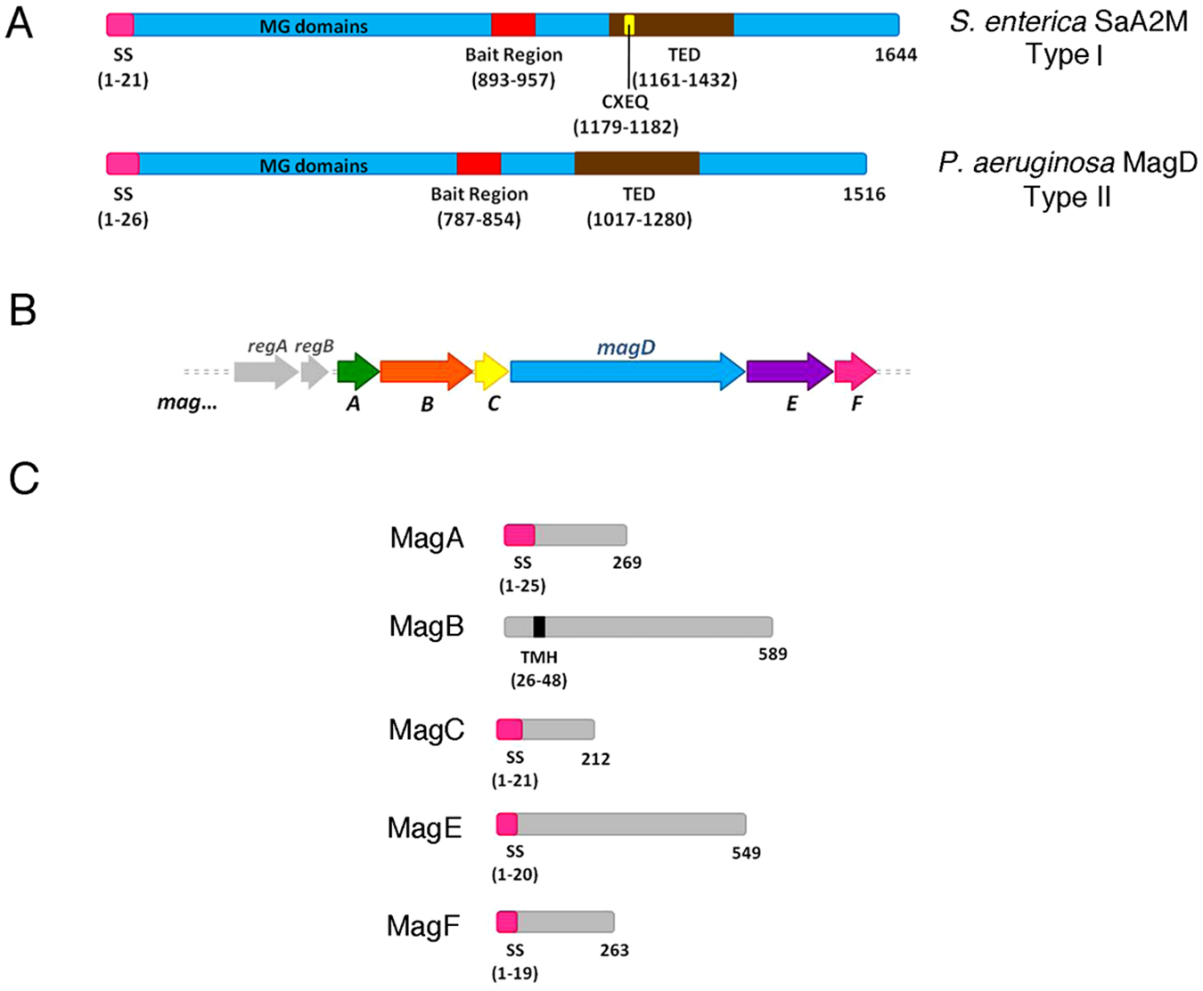
Schematic diagram of MagD, its partners, and its genomic environment. (**A**) Scheme of MagD (Type II) compared to Sa-A2M from *Salmonella enterica* (Type I). Both macroglobulins carry bait regions, but only SaA2M displays the conserved CXEQ motif required for the covalent bonding of proteases. (**B**) Genetic organization of *magD* within the six-gene Mag operon: *magA*, *magB*, *magC*, *magD*, *magE* and *magF*. (**C**) Schematic diagram of all Mag proteins encoded by the *mag* operon. SS, signal sequence; MG, macroglobulin; TMH, predicted transmembrane helix; TED, thioester domain.

In spite of the fact that molecules of the A2M/complement superfamily were believed to be limited to metazoans, genomic analyses revealed that genes for A2M-like proteins also exist in several bacterial species, many of which are pathogenic or are common colonizers of higher eukaryotes^7^. This calls into question the reason for the existence of molecules that resemble eukaryotic innate immunity proteins in prokaryotes. Two forms of A2M-like proteins were identified, only one of which contains the hallmark CXEQ thioester motif. Some bacteria, such as *Escherichia coli*, carry both forms of A2M, while others, such as *Salmonella enterica* or *Pseudomonas aeruginosa*, carry only one form (Fig. 1A)^7^. In a number of bacteria, the gene for the thioester-containing A2M (Type I) is adjacent to that encoding Penicillin Binding Protein 1c (PBP1c)^8^, a membrane-associated protein involved in cell wall synthesis. PBPs are involved in the biosynthesis of peptidoglycan (PG), a three-dimensional cross-linked mesh that surrounds the bacterium, giving it its shape and protecting it from differences in osmotic pressure^9^. PBPs are responsible for the two last reactions in PG biosynthesis, and thus play a key role in its stabilization^10^. The observation of the juxtaposition of *a2m* and *pbpC* genes led to the hypothesis that the two encoded proteins could work conjunctively as a periplasmic defense system. Thus, in the event of a cell wall breach, A2M would inhibit proteases and endopeptidases that penetrate the periplasm while PBP1c would act by repairing damage done to PG destroyed by lysozyme or other PG-targeting factors^7^.

We recently reported the crystal structure of a Type I A2M in three forms^11^, which revealed a fold and activation mechanism that are similar to those of eukaryotic complement proteins^12^. *S. enterica* A2M, a 180 kDa molecule consisting of 13 domains whose overall structure is reminiscent of that of eukaryotic C3, displays an exposed bait region and an entrenched CXEQ motif. Protease recognition occurs through specific targeting of the bait region, as is the case for eukaryotic forms. Garcia-Ferrer and co-workers^13^ and Fyfe and co-workers^14^ investigated protease recognition of the *E. coli* Type I A2M using electron microscopy and X-ray crystallography, and identified that upon protease binding, the macroglobulin undergoes a major conformational change; details of protease entrapment, however, could not be obtained since the target protease could not be visualized in electron density maps. The atomic details that describe a complex between a bacterial A2M and a protease are thus still an open question.

The second class of bacterial A2M (Type II), expressed by pathogens such as *P. aeruginosa*, lacks the CXEQ motif (but still carries the bait region) and is encoded within a six-gene operon (Fig. 1B). Recently, we showed that *P. aeruginosa* A2M (MagD) is a periplasmic protein associated preferentially with the inner membrane, and it is expressed by a majority of clinical strains^15^. MagD was shown, using immunoprecipitation pull-down assays in *P. aeruginosa*, to be associated to at least three other proteins from the *mag* operon (Fig. 1C): MagA, MagB and MagF. MagB is of particular interest, since it was predicted to carry a transmembrane helix^15^. Notably, the *magABCDEF* operon is co-expressed with operons encoding the Type VI secretion system as well those involved in exopolysaccharide biosynthesis, required for biofilm formation^16,17^. Moreover, strains lacking MagD display less virulence in an animal model of chronic infection^18^. These findings indicate that bacteria could employ Type II A2Ms to inhibit host proteases during infection, facilitating colonization of eukaryotic hosts, or block other bacterial proteases during microbial warfare, allowing survival in conditions where resources are scarce. How Type II A2Ms, which lack the thioester motif, can still entrap proteases, and what are the roles played by the other proteins expressed in the same operon are still unclear.

In this work we have addressed the structural and functional characterization of MagD as the central member of a multi-partite complex formed with other proteins encoded within the *mag* operon. We show that MagD can be actively recognized and cleaved by a specific protease within its bait region, an event which traps the protease in a stable complex, attesting to the ability of Type II A2Ms to display macroglobulin-like activity even in the absence of the classical thioester motif. In addition, we show by analytical ultracentrifugation (AUC) and small angle scattering (SAXS) that MagD is able to bind directly to MagA, MagB, and MagF with 1:1 stoichiometry. Furthermore, the formation of higher order complexes *in vitro* (MagD-MagA-MagB; MagD-MagF-MagB) requires the presence of MagB, the only protein predicted to carry a transmembrane domain. Notably, the absence of *magB* in *P. aeruginosa* results in the degradation of MagD in the bacterial periplasm, pointing to the key role MagB plays in complex formation. Type II A2Ms are thus atypical members of a rudimentary bacterial immune system that protects the periplasm by employing a multi-partite complex whose central member structurally mimics eukaryotic immune proteins.

## Results

### MagD can entrap a specific protease non covalently

Although MagD does not carry the CXEQ thioester motif involved in the covalent association to target proteases (Fig. 1)^11,13,14^, we reasoned that if its biological role is linked to protection of the cell from protease attack, it should be targeted by different proteases as shown for Type I A2Ms^19^. We initially performed proteolysis tests of MagD using chymotrypsin, trypsin and papain, and analyzed results by SDS-PAGE, N-terminal sequencing and mass spectrometry (Fig. S1). In all three cases, the bait region was cleaved, generating two species that remained associated to each other and migrated together as a single peak in gel filtration. Notably, the first 198 amino acids, which are presumably flexible, were also proteolyzed.

**Figure 2:**
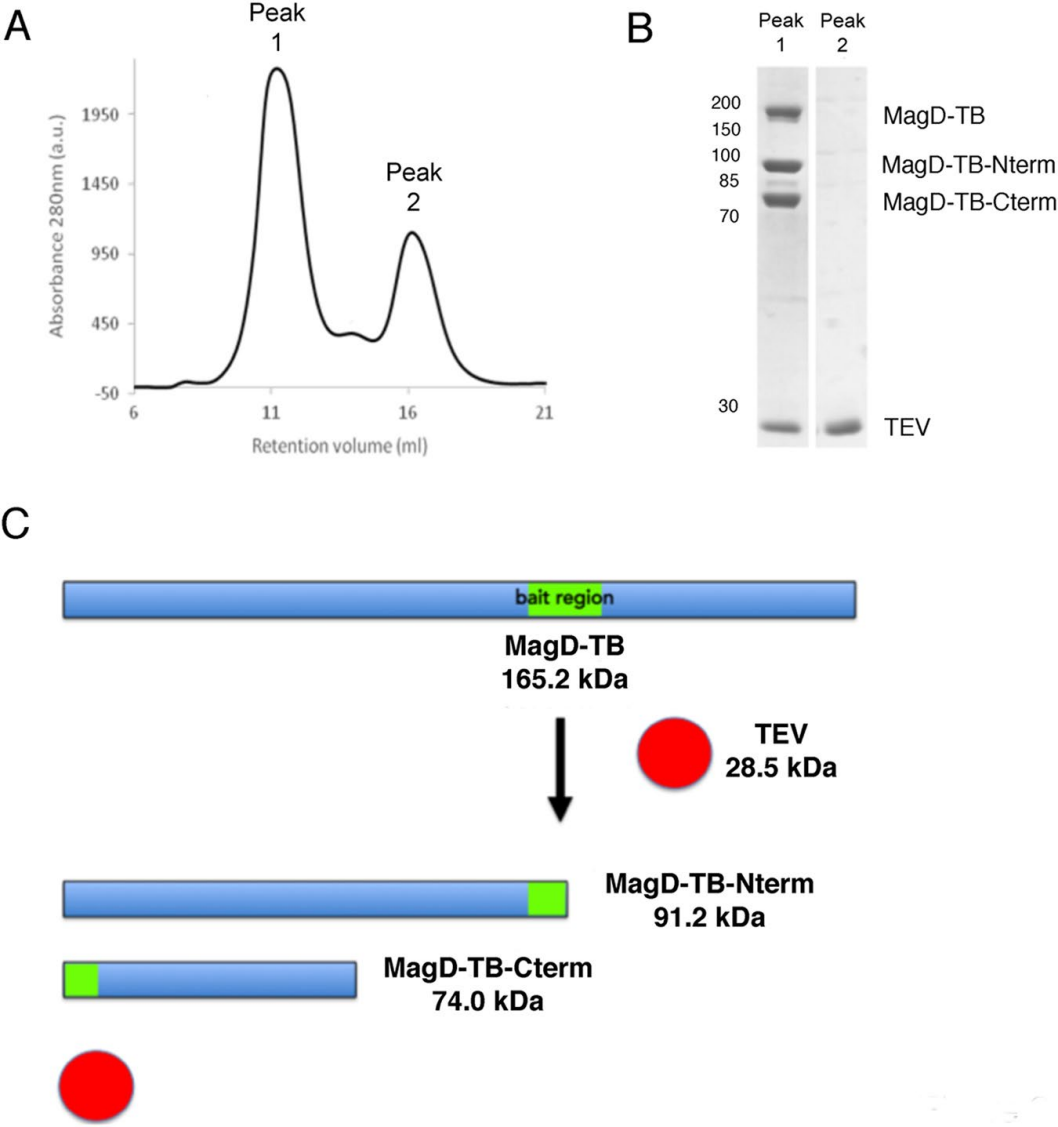
MagD displays macroglobulin-like protease trapping activity. (**A**) Incubation of MagD-TB with TEV followed by gel filtration on a Superdex 200 10/30 column leads to the appearance of a peak that contains four bands, as shown on the gel in (**B**): uncleaved MagD-TB, two forms of MagD-TB cleaved at the bait region, and TEV. Positions of molecular mass markers are indicated on the left. The gel includes only lanes of interest for analysis of the result. (**C**) Schematic diagram of the reaction that occurs upon MagD-TB recognition by TEV.

In order to study MagD targeting by a protease that would only recognize the bait region, we designed a mutant form by introducing a recognition sequence for the TEV protease onto the predicted MagD bait region, as previously done for the study of the A2M from *Salmonella enterica*^11^; we called this mutant MagD-TB (for ‘TEV Bait’). The MagD bait sequence ANRSERG (amino acids 847–853) was substituted by ENLYFQG, which if recognized and cleaved by TEV would generate two distinct fragments of 93 kDa (N-term) and 74 kDa (C-term). Apo MagD-TB was incubated overnight with an excess of TEV and subsequently subjected to SEC analysis (Fig. 2). SDS-PAGE analysis of the resulting elution peak indicated 4 bands: uncleaved (full-length) MagD-TB, TEV, and two intermediary forms which were shown by N-terminal sequencing and mass spectrometry to correspond to the expected 91.2 kDa and 74.0 kDa fragments of cleaved MagD-TB (Table 1). These results thus confirmed that despite the absence of the CXEQ motif, MagD is still able to trap target proteases following a bait region-cleaving mechanism in which the protease remains non-covalently associated to MagD. This is a unique characteristic of Type II A2Ms, which leads to the suggestion that protease entrapment occurs through a mechanism that is distinct from what has been proposed to date for most eukaryotic or prokaryotic macroglobulins.

**Table 1:** Mass spectrometry analyses of MagD/TEV incubation experiment.

Band (Fig. 2B)	Expected mass (Da)	Observed mass (Da)	Mass deviation (Da)	Identification
1	165,232.78	165,241.69	8.2	MagD (2-1495), full-length
2	91,231.90	91,234.90	2.9	MagD (2–821) N-terminal fragment
3	74,018.90	74,020.88	1.9	MagD (882–1495) C-terminal fragment
4	28,556.42	28,556.55	0.24	TEV protease

### MagD interacts individually with MagA, MagB, and MagF forming 1:1 complexes

Previously, we had shown that MagA, MagB, MagD and MagF co-immunoprecipitate from the inner membrane of *P. aeruginosa*^15^. MagA, MagB, and MagF are considerably smaller than MagD (26–60 kDa *versus* 165 kDa) and carry signal peptides at their N-termini (except for MagB; Fig. 1C). In order to initiate the characterization of this multi-partite complex, we expressed MagA, MagB, MagD and MagF in the *E. coli* cytoplasm, but all proteins, with the exception of MagD, were expressed as inclusion bodies. We thus tested co-expression of MagD with MagA, MagB or MagF, and this strategy allowed the co-purification of soluble binary complexes, indicating that MagD is able to interact individually with the three Mag proteins in stable form (Fig. S2). We employed the same strategy to test binary complex formation between MagD and MagC and MagE, but could not show stable complexes by gel filtration chromatography.

**Figure 3:**
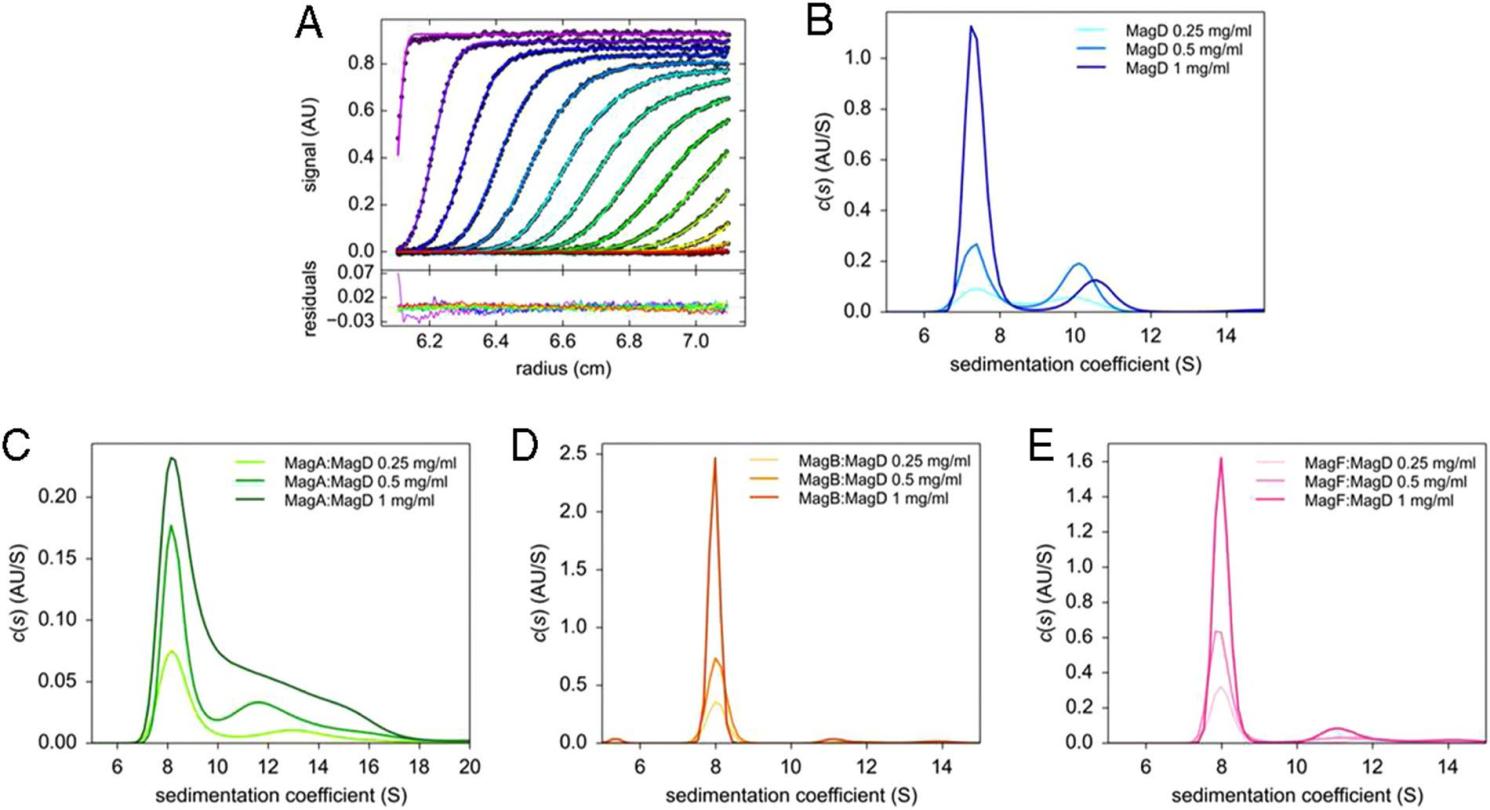
Sedimentation velocity of MagD and Mag binary complexes (**A**) (Upper panel) Fitting of experimental sedimentation profiles of MagD using Sedfit and its residual (lower panel) (**B**) Results of the *c(s)* analysis for MagD (apoform), MagA:MagD (**C**), MagB:MagD (**D**), MagF:MagD (**E**) complexes. In (**B**), since the proportion of dimer does not increase with concentration, it probably does not result from a thermodynamic equilibrium. Samples were studied at 0.25, 0.5 and 1 mg/ml, at 20 °C with a rotor velocity of 35,000 rpm and an absorbance monitoring at 280 nm.

To investigate the stoichiometry of the binary Mag complexes, samples were studied by analytical ultracentrifugation using the sedimentation velocity method. After analytical gel filtration, MagA:MagD, MagB:MagD, and MagF:MagD samples were centrifuged at 35,000 rpm using a Beckman ultracentrifuge at 20 °C with absorbance monitoring at 280 nm. Consecutive scans were automatically recorded at regular intervals and analyzed with Sedfit using the continuous size distribution *c(s)* analysis method to determine sedimentation coefficients^20^. Purified MagD and Mag complexes were analyzed at three different concentrations: 0.25 mg/mL, 0.5 mg/mL and 1 mg/mL (Fig. 3). MagD sedimented as two species (at all three concentrations): a major form with a mean _S20,w_ value of 7.8 S +/− 0.5 S, compatible with a monomer, and a minor form presenting a mean _S20,w_ value of 10.3 S +/− 0.6 S, which is compatible with a dimer (Table 2). The MagA:MagD, MagB:MagD, and MagF:MagD complexes sedimented with mean _*S*20,w_ values of 8.7 S, 8.4 S and 8.3 S, that are slightly higher than that of MagD and which indicate stoichiometries of 1:1 for the complexes (Table 2). Notably, the MagB:MagD and MagF:MagD sedimentation peaks were sharp, indicating rather homogeneous complexes, but the MagA:MagD peak displayed a clear shoulder indicating the presence of a mixture of higher order species (Fig. 3C). Nevertheless, these data clearly indicate that MagD can form stable 1:1 complexes with these three specific Mag partners.

**Table 2:** Mass of Mag complexes obtained from analytical ultracentrifugation.

Sample	Theoretical mass (approx. kDa)	_*S20,w*_ value	Fitted mass (kDa)
MagD	165	7.8 S +/− 0.5 S 10.3 S +/− 0.6 S	160 +/− 18 246 +/− 28^a^
MagA:MagD	192	8.7 S +/− 0.6 S	192 +/− 16
MagB:MagD	225	8.4 S +/− 0.3 S	212 +/− 3
MagF:MagD	191	8.3 S +/− 0.3 S	189 +/− 18

^a^Fitted globally.

**Figure 4:**
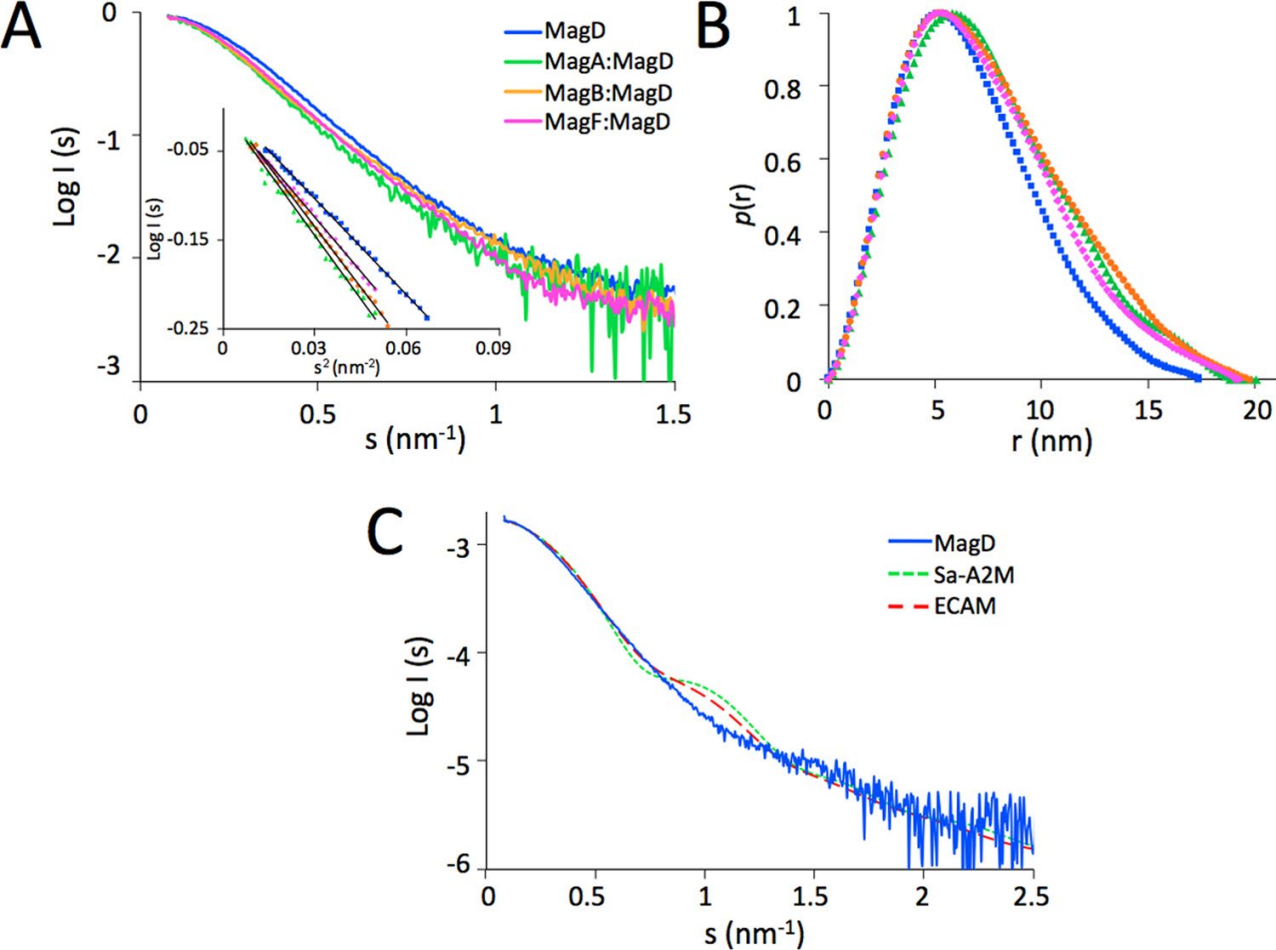
Small angle X-Ray Scattering of MagD and Mag binary complexes. (**A**) Experimental data: scattering curves are presented in the form log I versus s (nm^−1^) where I is the scattering angle. Scattering data for MagD in native form (blue), in complex with MagA (green), MagB (orange) and MagF (magenta) were recorded at concentrations of 2 mg/mL for MagA:MagD and 6 mg/mL for the others. The inset presents the Guinier plot. Curves were normalized to have a *I*(0) = 1. (**B**) Pair-distance distribution function *p*(r) of the same data. (**C**) Measured scattering curve of MagD (solid blue) compared to the calculated scattering curves of *S. enterica* (dotted green, PDB 4U48) and *E. coli* (dashed red, PDB 5A42) A2M, calculated with CRYSOL.

We then sought to further characterize the binary MagD complexes by using small angle X-ray scattering (SAXS). Experiments were performed with MagD, MagA:MagD, MagF:MagD, and MagB:MagD that had been purified by size exclusion chromatography prior to experimentation. Data were collected at the D01B-SAXS1 beamline at the LNLS light source in Brazil (Fig. 4A and Table 3). Scattering patterns, recorded at different concentrations for all samples, did not suggest any oligomerization or aggregation events. The Guinier plots were linear, and data at low angles did not indicate any aggregation.

**Table 3:** Data collection and structural parameters of the Mag complexes obtained using SAXS. Rh and Rg values obtained from SEC and DLS were also included for comparative purposes.

	MagD	MagA:MagD	MagB:MagD	MagF:MagD
**Data collection parameters**				
Instrument	LNLS-SAXS1 beamline			
Beam geometry	Pinhole geometry, Pilatus 300 k			
Wavelength (Å)	1.544			
*s* range (nm)	0.068–2.8			
Exposure time (sec)	100			
Temperature (°C)	10			
Concentration range (mg/mL)	2–6			
**Structural parameters** ^1^				
*R*_*g*_ (nm) (from Guinier)	5.0 ± 0.1	5. 8 ± 0.2	5.6 ± 0.1	5.3 ± 0.3
*R*_*g*_ (nm) (from (*P(r)*)	5.2	5.9	5.9	5.7
*I(0)* (cm^−1^) (from Guinier)	0.0018 ± 3.8 10^−6^	0.00059 ± 3.1 10^−6^	0.0019 ± 5.4 10^−6^	0.0022 ± 6.4 10^−6^
*D*_max_ (nm)	17.6	20.1	19.8	19.3
Rh from SEC (nm)	5.1	5. 3	5.2	5.2
Rh from DLS (nm); polydispersity (%)	5.1 (7.4)	5.3 (10.0)	5.2 (9.7)	5.2 (9.2)
**Software employed**				
Primary data reduction	FIT2d			
Data processing	PRIMUS/GNOM			

^1^Reported for the 6 mg/mL measurements, except for MagA:MagD, at 2 mg/mL.

Dmax (nm) (+/−0.5 nm).

Rh from SEC (nm) (+/−0.5 nm).

Rh from DLS (nm) (+/−1 nm).

The structure of apo MagD is reminiscent of that of Type I bacterial macroglobulins^11,13,14,21^. Indeed, when comparing the MagD diffusion curve to those of *E. coli* A2M (ECAM; PDB 5A42)^13^ or *S. enterica* A2M (Sa-A2M; PDB 4U48)^11^, which were calculated from their structures using CRYSOL^22^ (Fig. 4C), it is clear that the three diffusion curves fit well at small angles, indicating similar overall dimensions. At higher angles, the fits are less good, and could be related to different relative positions of the two N-terminal domains that appear to be flexible when comparing ECAM and Sa-A2M. We note that the Rg value measured here for MagD is slightly larger than the one measured previously^15^.

The Guinier analyses showed an increase in the radii of gyration (Rg) value when comparing MagD and the binary complexes (Fig. 4A inset and Table 3). Slightly larger dimensions for the complexes are corroborated by the values for Rg and calculated from the *p*(r) distributions curves. The maximum particle dimension (Dmax) and Rg values for all bipartite complexes, with Dmax values ranging from 19.3 nm (MagF:MagD) to 20.1 nm (MagA:MagD) and Rg values ranging from 5.7 to 5.9 nm were higher than those for native MagD (Dmax = 17.6 nm, Rg = 5.2 nm) (Table 3). Thus, the modification in Dmax and Rg values, as well as the change in the shape of the *p*(r) curve (Fig. 4B), indicate that there is an increase in measured dimensions when MagD binds to any of its partners.

SAXS experiments were complemented by SEC and Dynamic Light Scattering (DLS) measurements. The comparative analysis of Rg values obtained from SAXS experiments and Rh values calculated from analytical SEC and DLS showed the consistency of these data, which pointed to a slight increase of MagD dimensions in the presence of its partners (Table 3).

**Figure 5:**
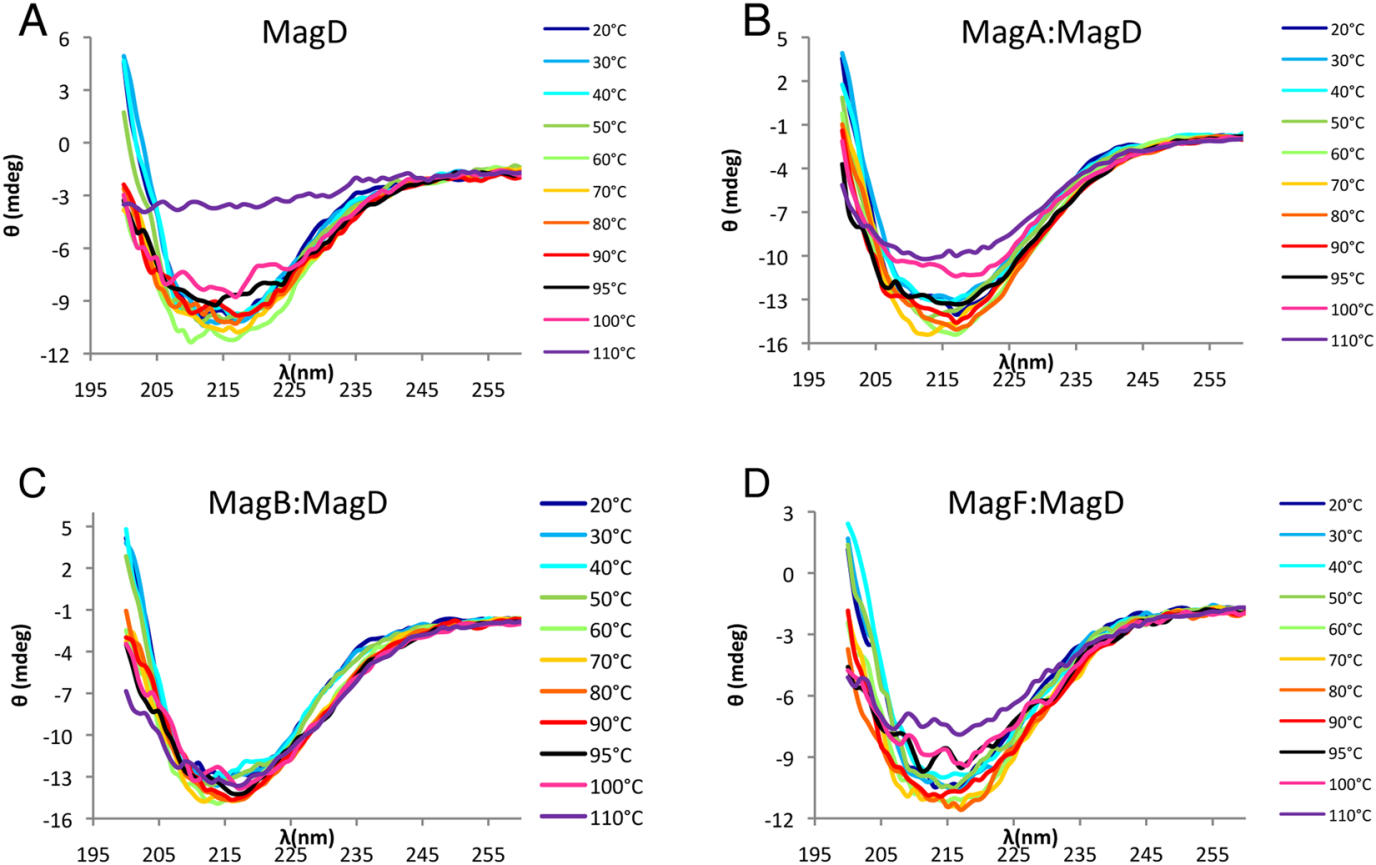
Thermal unfolding of MagD and Mag binary complexes. CD spectra were recorded from 260 to 200 nm using a temperature ramp of 20 °C to 110 °C and using an increment rate of 1 °C/min. Protein samples were tested at 0.5 mg/ml, in a buffer containing 10 mM HEPES pH 7.5 and 40 mM NaCl. For clarity, only spectra recorded at 20, 30, 40, 50, 60, 70, 80, 90, 95, 100 and 110°C are presented.

To characterize the stability of the Mag complexes, we monitored the thermal unfolding of the samples using circular dichroism. Scans were performed in the 260 nm–200 nm range every 1 °C between 20 °C and 110 °C (Fig. 5). Thermal denaturation measurements showed that MagD conserves its secondary structure up to 95 °C, after which it unfolds rapidly. Thermal stability is improved when MagD is complexed to one of its partners. MagA:MagD and MagF:MagD samples still displayed secondary structure even at 110 °C. Interestingly, the MagB:MagD complex displayed the highest stability, conserving a constant minima at 216 nm even at 110 °C. These data suggested that MagA, MagF, and MagB stabilize the structure of MagD, with MagB displaying the highest capacity for complex stabilization.

### MagB plays a key role in complex stabilization in a cellular context

**Figure 6:**
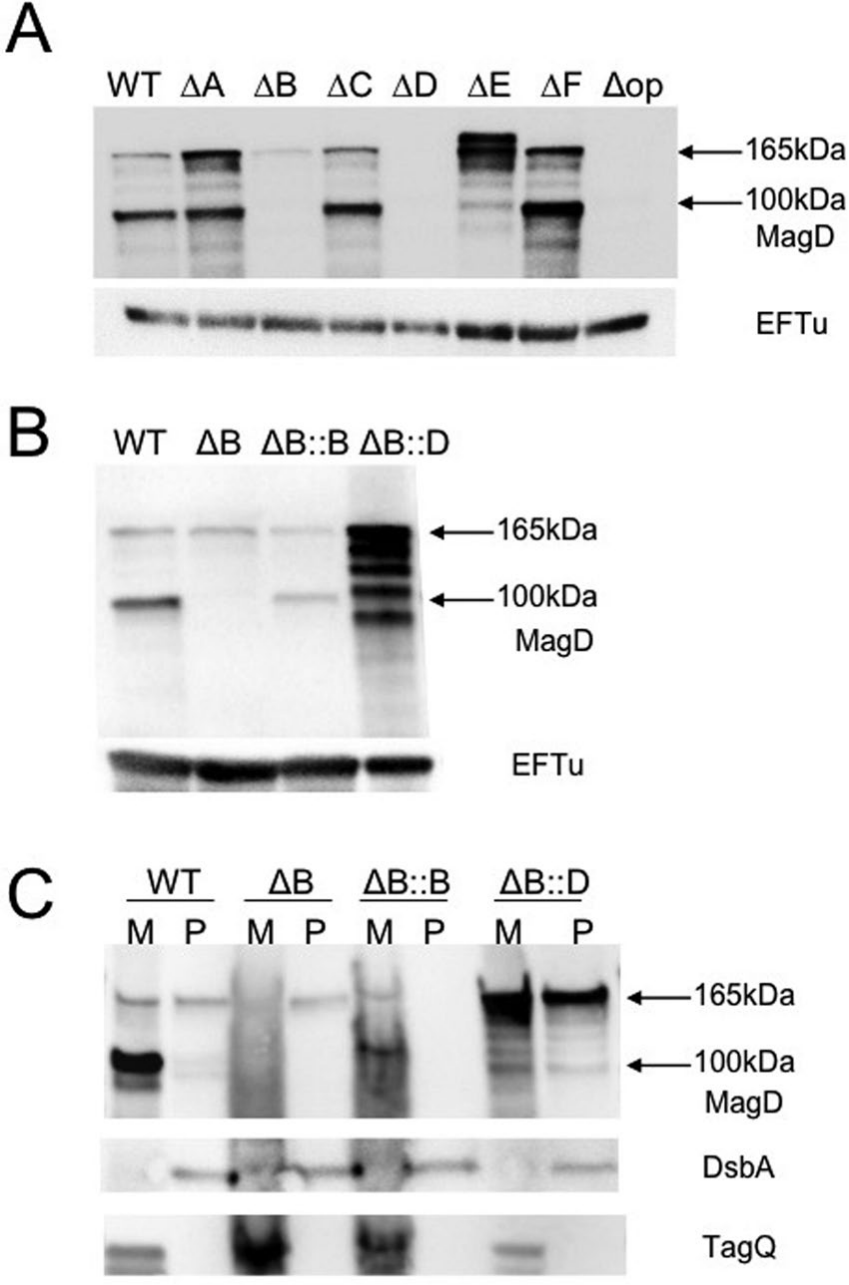
MagD stability and cleavage are affected in mutants lacking MagB and MagE. (**A**) Crude extracts of *P. aeruginosa* PAO1 (WT) or knock-out strains lacking indicated proteins were run on SDS-PAGE and immune-developed with polyclonal anti-MagD antibodies. The Δop mutant^15^ that lacks the entire *mag* operon was used as control. In the wild-type background MagD appears as 165 kDa native and 100 kDa cleaved forms. The 100 kDa form is absent in Δ*magB* and Δ*magC* backgrounds. An additional higher molecular weight form observed in *ΔmagE* could correspond to unprocessed MagD still containing the signal peptide (*). (**B**) Crude extracts of indicated strains lacking MagB (Δ*magB*) or expressing *magB* or *magD* in trans were analyzed as in (**A**). (**C**) Bacteria were further fractionated into membranes and periplasm showing that MagB governs macroglobulin membrane localization and cleavage to the 100 kDa form. Note the presence of 165 kDa form of MagD exclusively in the periplasm of the *magB* mutant. This form is subject to rapid degradation as visualized by products of smaller sizes in the Δ*magB*::*magD* strain. In the Δ*magB*::*magB* strain, membrane localization was restored and MagD appears as 165 kDa and 100 kDa forms, similarly to wild-type. EFTu, DsbA and TagQ antibodies were used as loading controls for whole bacterial extracts, periplasm and membrane samples, respectively. Gels have had lanes selected/cropped for clarity.

We have previously shown that the deletion of the whole *mag* operon (encompassing *magABCDEF*) had a dramatic effect on MagD, influencing its localization, stability and cleavage from the full-length, 165 kDa form to a 100 kDa form^15^. To confirm the key role of MagB identified by our *in vitro* assays and to delineate the specific role of each of the other Mag proteins, we individually deleted each gene from the *mag* operon and investigated the fate of MagD by Western blotting using anti-MagD antibodies (Fig. 6A). Deletions of *magA*, *magC* and *magF* had no pronounced effect on MagD, since we could detect the protein in its two forms (165 and 100 kDa) in similar ratio/quantities, albeit with higher amounts of the full-length form in *ΔmagA*. This result is probably related to the increased stability levels of the whole RmsA-regulated mRNA in the strain carrying the *magA* deletion. However, the absence of MagB and of MagE greatly influenced the amount of MagD; only a barely detectable amount of the non-cleaved 165 kDa form could be visualized in the *magB* deleted strain. Importantly, the 100 kDa form was undetectable. This indicates that MagB could play a direct role in MagD stabilization and could be involved in its cleavage to the 100 kDa form. MagB expression *in trans* in a Δ*magB* mutant restored the presence of the two forms. Interestingly, expression of MagD in trans in a Δ*magB* background resulted in higher quantities of MagD but with prominent degradation (Fig. 6B), an observation that was also made for MagD in the Δ*OP* background^15^.

It is of note that in a mutant lacking *magE*, additional forms of higher molecular mass were detected. Our previous work suggested that MagE is cytoplasmic and does not associate stably with the Mag complex^15^. Therefore, MagE could be involved in cleavage of the signal peptide from MagD, allowing its translocation from the cytosol to the periplasm. The higher forms of MagD could thus correspond to its unprocessed, full-length form still harboring the 21 residue signal peptide, as well as degradation products.

### The inner membrane localization of MagD and its cleavage are determined by MagB

When full-length MagD was expressed *in trans* in a strain where the entire *mag* operon was knocked out (*P. aeruginosa* Δop), it lost its inner membrane localization^15^. Since MagB is the only protein encoded by the Mag operon harboring a predicted transmembrane domain (amino-acids 26–48) and it plays an important role in MagD stabilization in both *in vitro* and *in vivo* assays, we investigated if it could play a direct role in MagD localization. We thus performed localization studies of MagD in a Δ*magB* strain with or without MagB supplemented *in trans*. To that aim, *P. aeruginosa* total membranes and periplasm were separated and immunoblotted using anti-MagD antibodies. As shown in Fig. 6C, in the strain deleted for *magB*, the majority of full-length (165 kDa) MagD was found in the bacterial periplasm, while expression *in trans* of MagB restored its membrane localization. Moreover, the 100 kDa form could be readily detected in the complemented strain. These results show that MagB dictates the inner-membrane localization of the Mag complex, which directly or indirectly results in MagD processing into a 100 kDa form. In the same manner, we isolated membranes and periplasm in a Δ*magB* strain overexpressing *magD in trans*; we found a fraction of full length MagD in membranes and again a fraction of the protein in the periplasm. Degraded forms could be observed in both bacterial compartments. These results suggest that all Mag proteins must be co-expressed from the same operon in similar amounts for optimal complex formation, possibly due to a highly regulated partner association/localization mechanism.

### MagB is a key member of a higher ordered Mag complex

**Figure 7:**
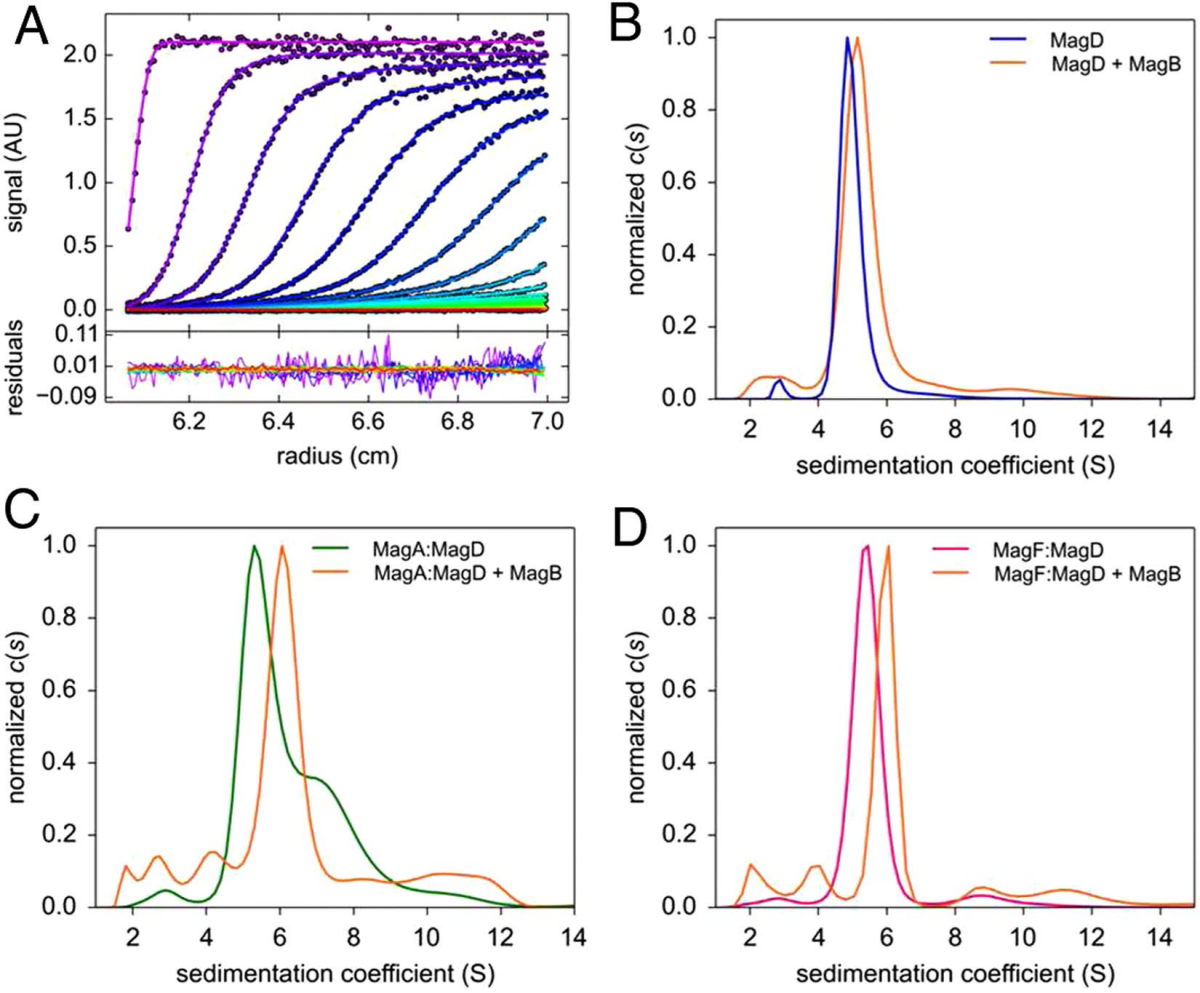
Sedimentation velocity of ternary Mag complexes. (**A**) (Upper panel) Fit of experimental curves of MagD + MagB sedimentation using Sedfit and its residual (lower panel). Results of the c(s) analysis for (**B**) MagD + MagB, (**C**) MagA:MagD + MagB, (**D**) MagF:MagD + MagB. Samples were studied at 0.5 mg/ml, at 20 °C with a rotor velocity of 35,000 rpm and monitoring the absorbance at 280 nm. The buffer included 5% glycerol. Note that addition of MagB to the MagA:MagD sample causes disappearance of the shoulder at approximately 7 S, suggesting stabilization of a unique ternary form.

As mentioned above, MagB, when expressed alone, is largely localized to inclusion bodies, but a small amount of soluble protein could be isolated and purified. To further investigate the role of MagB in Mag complex formation, we incubated purified MagB with MagA:MagD and MagF:MagD as well as MagD alone and performed AUC measurements (Fig. 7). The MagB sedimentation profile showed a major peak at _*s20,w*_ of 3.3 S, corresponding to the monomeric form of the protein (not shown). Upon incubation of MagB with MagA:MagD, the sedimentation peak of the binary complex shifted from an _*s20,w*_ value of 6.6 S to 7.3 S (Fig. 7C), indicating the formation of a stable tripartite complex. Similarly, incubation of MagB with MagF:MagD also yielded a shift in the complex sedimentation peak from _*s20,w*_ of 6.5 S to 7.1 S (Fig. 7D). These results demonstrate that MagB is able to stably interact with MagD once it is already in complex with either MagA or MagF, forming the ternary complexes MagB:MagA:MagD and MagB:MagF:MagD. This also indicates that the binding sites for MagB, MagA, and MagF on the surface of MagD do not overlap.

## Discussion

A2Ms are key proteins of the eukaryotic innate immune system, and the identification of bacterial genes encoding A2M-like proteins suggested that bacteria could also employ macroglobulin-like molecules in infectious or colonization processes^7^. The hallmark of A2M action is a CXEQ thioester motif, buried deep within the structure in order to protect it from hydrolysis, which is activated once the target protease cleaves the bait region, thus becoming covalently trapped^3^. The identification of bacterial A2M-like proteins that harbor bait regions but not CXEQ motifs (Type II A2Ms) put forth the question of the activity, and biological role, of such molecules. It is of interest that Type II A2Ms are often encoded in a six-gene operon, a situation which is quite distinct from that of Type I molecules (CXEQ-carrying), which are encoded in an operon that co-expresses the peptidoglycan synthase PBP1c.

We had previously structurally and functionally characterized a Type I A2M, Sa-A2M, which, in absence of a protease substrate, guarantees the stability of its thioester site through the action of a “lock”. Once the protease-A2M reaction has taken place, Sa-A2M is cleaved into two products (102 kDa and 77 kDa), with the protease remaining covalently associated to the smallest form. An Sa-A2M mutant in which the thioester was inactivated was unable to trap the target protease^11^. The Type II A2M characterized in the present work (MagD) does not carry a thioester region, and yet upon incubation with a specific protease (in this case, TEV), the bait region was cleaved and the protease was stably trapped, as shown by gel filtration, N-terminal sequencing, and mass spectrometry experiments (Fig. 2). The TEV protease cleavage of MagD resulted in the generation of two products: 91.2 kDa (N-terminal) and 74.0 kDa (C-terminal). It is of note that despite the large excess of TEV, MagD was not fully cleaved, whether in its apoform or in the presence of MagA, MagB and MagF respectively (data not shown). This could indicate that the Mag complex must be fully assembled, with the presence of all partners, in order for full activation to be achieved. Thus, similarly to Sa-A2M and its “locked” conformation, MagD could possess an “inhibited” conformation where the bait region is not completely accessible. This assumption is supported by the observation that *in vivo*, MagD can be cleaved to a 100 kDa form only under specific conditions, i.e., in the presence of MagB. This suggests that Type II A2Ms have somehow evolved to be more highly regulated than their Type I counterparts in protease-recognition and trapping activities, a fact which is potentially linked to the circumstances in which they are expressed (i.e., during infection or colonization processes^16–18^).

Limited proteolysis experiments performed with MagD indicated that in addition to the bait region, MagD was cleaved between Tyr163 and Asp164, as well as Lys198 and Leu199. These first 199 residues, which are potentially flexible and thus more easily accessible to proteases, correspond to the two initial MG domains of the Type I homologs SaA2M and ECAM, whose structures indicated high flexibility for this region^11,13,14,21^. In ECAM, these domains have been suggested as being implicated in its anchoring to the inner membrane^19^. MagD, however, does not carry a specific lipobox region or a predicted transmembrane domain; nevertheless, it associates with the inner bacterial membrane, and its presence is necessary for stability of the entire Mag complex in bacterial cells^15^. Here, we show that it is the 589-residue MagB that is specifically responsible for associating MagD, and thus the entire Mag complex, to the inner membrane. In addition, MagB plays a key role in structural stabilization of the complex, as seen in our CD results (Figs 6 and S3).

**Figure 8:**
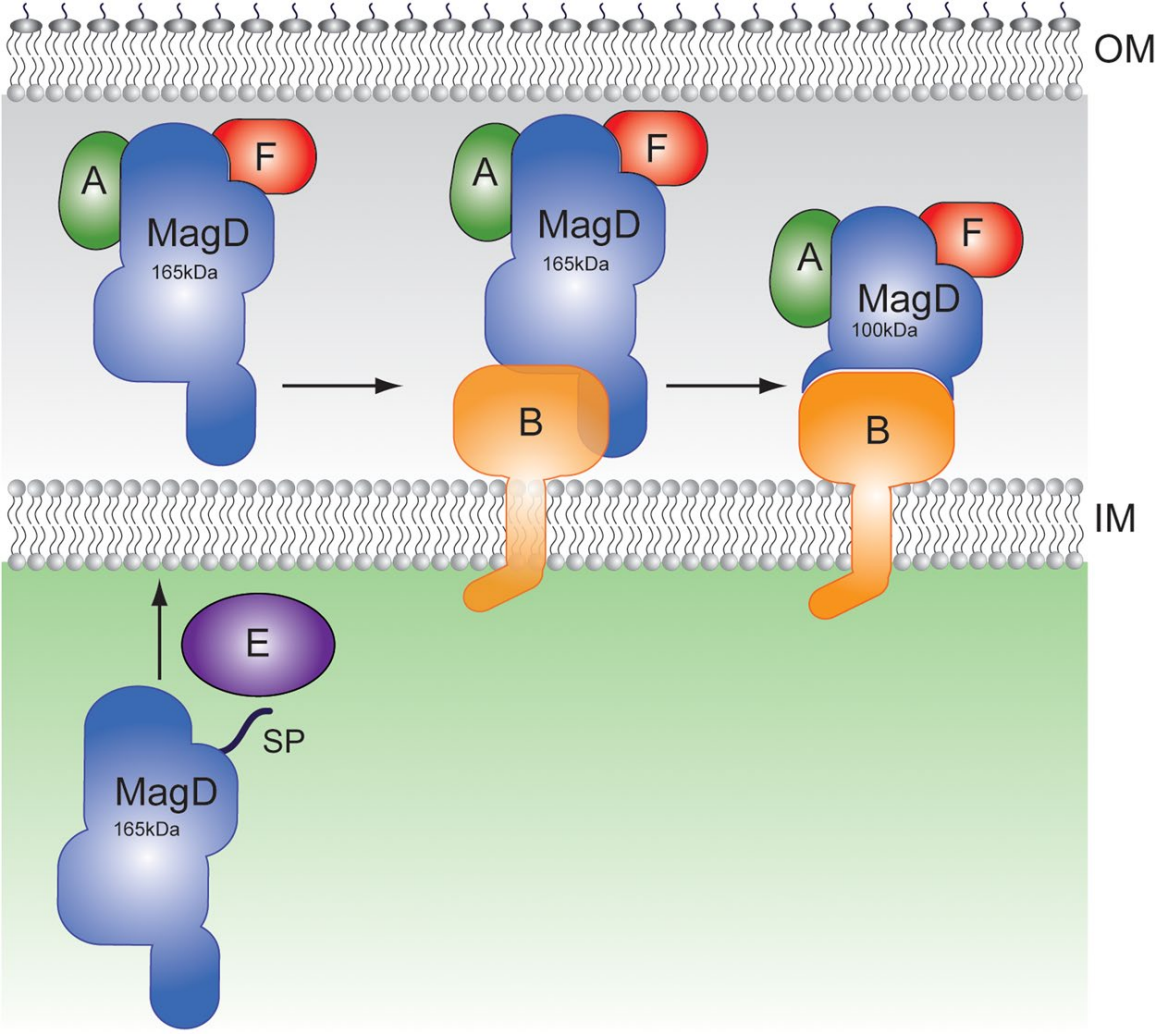
Model of Mag complex formation on the bacterial periplasm. MagB plays a key role in the stabilization of the complex; MagD can bind to MagA and MagF in both of its forms (165 and 100 kDa). MagE is potentially involved in cleavage of the signal peptide of MagD. IM, inner membrane; OM, outer membrane.

MagB is able to associate to binary complexes MagA:MagD and MagF:MagD, comforting the idea that the binding sites for the partner proteins are not overlapping on MagD. Unfortunately, due to solubility issues regarding MagA and MagF, we were not able to test if these isolated molecules could simultaneously bind to the isolated MagB:MagD binary complex. However, the identification of stable interactions between MagA, MagB, MagD and MagF clearly indicate the formation of ternary complexes, with a potential for formation of higher order complexes. Independent of the stoichiometry of the studied complexes, MagB plays a clear, central role. This could also be observed in experiments performed directly on *P. aeruginosa* cells (Fig. 6). We show that MagB is essential for the cleavage of the full-length 165 kDa form to a 100 kDa form, previously suggested as representing a potential direct or indirect activation process^15^. Interestingly, the deletion of *magE* seems to prevent MagD from being processed. It is thus conceivable that MagE could play the role of chaperone in order to facilitate MagD’s transport across the inner membrane and/or processing by a signal peptidase. Figure 8 depicts a model for Mag complex formation that summarizes these results.

The question remains, however, as to why bacteria need to carry a rudimentary immune system with two types of macroglobulins, large, multi-domain molecules. The Type II macroglobulin characterized in this work also requires association to additional proteins on the cell membrane for optimal functioning and stabilization (Fig. 8), and generation of such an assembly is potentially energetically costly for the bacterial cell. It is of note that Mag complexes are also highly stable, with different Mag partners remaining associated even when MagD is cleaved to its 100 kDa form (Fig. 8). The widespread presence of these genes in pathogens and colonizers, as well as the link that has been identified with diminished virulence and the Type VI secretion system, indicate that bacterial macroglobulins could play key roles in fitness during infection, antimicrobial warfare, and/or colonization. In *P. aeruginosa*, it is conceivable that the Mag complex participates in defense of the bacterial periplasm during these processes, with MagD playing the role of protease entrapment and other Mag proteins providing stabilization and/or regulation for the complex.

## Materials and Methods

### Cloning and protein purification

The *magD* gene was cloned into the pETDuet-1 vector for protein overexpression in *Escherichia coli* BL21 Star in such a way that the final expressed product would carry a hexa-histidine sequence at its N-terminus, in lieu of the first 37 amino acids (corresponding to the signal peptide and lipobox). Bacteria were grown in LB medium containing 100 µg/mL of ampicillin at 37 °C. At mid-exponential growth (OD_600_ 0.6–0.8), 0.5 mM of IPTG were added to induce expression during 4 h at 30 °C. The cells were harvested by centrifugation at 5,500 rpm for 20 min at 4 °C. The cell pellet was resuspended in lysis buffer (50 mM HEPES pH 7.5, 300 mM NaCl and 20 mM imidazole). A commercial mixture of protease inhibitors (SIGMAFAST^TM^, Sigma-Aldrich) was added to the suspension, which was then sonicated, and the cell lysate was centrifuged at 20,000 *g* for 45 min at 4 °C. The supernatant was filtered (0.20 µm) and loaded onto a Ni^2+^-NTA column equilibrated in lysis buffer. After extensive washing, the His_6_-tagged protein was eluted with a gradient of imidazole to 300 mM. The eluate was concentrated and injected onto a size exclusion chromatography column (Superdex S200 16/60, GE Healthcare) equilibrated in 25 mM HEPES pH 7.5, 100 mM NaCl. The purification was performed at 4 °C on an HPLC ÄKTA Purifier (GE Healthcare). The purified His_6_-tagged protein was concentrated by ultra-filtration (Vivaspin).

The synthetic *magA, magB magC, magE and magF* genes (Genscript) were cloned into pET28a. The sequence corresponding to the N-terminal signal peptides was replaced by a hexa-histidine tag sequence. In the case of *magB*, the predicted transmembrane helix was also deleted from the sequence. Co-expression of Mag complexes was carried out by transforming *E. coli* BL21 Star cells with both pETDuet-1-*magD* and pET28a-*mag* plasmids expressing any of the other Mag proteins. Bacteria were grown in LB medium containing 100 µg/mL ampicillin and 50 μg/mL kanamycin at 37 °C. At mid-exponential growth (OD_600_ 0.6–0.8), 0.2 mM of IPTG were added to induce expression overnight at 22 °C. The cells were harvested by centrifugation at 5,500 rpm for 20 min at 4 °C and complexes were purified using the same purification protocol as described above for MagD.

### MagD mutagenesis and TEV reaction

The MagD bait region sequence ANRSERG (comprising the residues 847–853) was converted into the ENLYFQG sequence, which is recognized by Tobaco Etch Virus (TEV) protease, by site-directed mutagenesis using primers designed with the NEBaseChanger tool (http://nebasechanger.neb.com/). Briefly, primers were phosphorylated and used for amplification. The reaction was then incubated with DpnI (Thermo/Fermentas) to eliminate the template and subsequently purified from an agarose gel. The final product was then ligated and transformed into MACH1-T1R competent cells, and clones were confirmed by sequencing. MagD was incubated with a large excess (a molar ratio of 1:2) of TEV protease in a buffer containing 25 mM HEPES pH 7.5, 100 mM NaCl, 1 mM DTT and 0.5 mM EDTA overnight at 4 °C. Samples were injected onto an analytical size exclusion column (Superdex S200 10/30) and analyzed by 12.5% SDS-PAGE.

### Dynamic Light Scattering

Measurements of all sample solutions were conducted using a Protein Solutions DynaPro DLS system (Wyatt). Protein samples ranging from 0.5 mg/mL to 1 mg/mL were measured in duplicate at 20 °C. One measurement corresponded to 20 acquisitions of 5 seconds.

### Circular Dichroism

Thermal unfolding was executed monitoring CD signal from 260 to 200 nm from 20 °C to 110 °C with a temperature rate of 1 °C/min. Measurements were performed on a Jasco J-810 Spectropolarimeter with Pelletier control using 1 mm Quartz cuvettes (Hellma) with a scan speed of 50 nm/min, 3 accumulations, and a response time of 4 seconds. Protein samples were tested at a concentration of 0.5 mg/ml in a buffer consisting of 10 mM HEPES pH 7.5 and 40 mM NaCl (protein buffer diluted 2.5 fold in water). Signals are reported as raw ellipticity and all the spectra were corrected for solvent contribution.

### Analytical ultracentrifugation

Sedimentation velocity experiments were performed in a Beckman Coulter OptimaTM XL-A (Beckman) ultracentrifuge at 20 °C. Samples were centrifuged at 35,000 rpm with absorbance monitoring at 280 nm. Protein concentrations ranged from 0.25 to 1.0 mg/mL and samples were prepared in 25 mM HEPES pH 7.5 and 100 mM NaCl (density 1.0043 g/mL and viscosity 1.029 cp). Experiments involving apo MagB (shown in Fig. 7) were performed with samples obtained in buffer containing 25 mM HEPES pH 7.5 and 100 mM NaCl supplemented with 5% glycerol where buffer density and viscosity values employed (but not measured, and perhaps imprecise) corresponded to 1.016 g/mL and 1.156 cp, respectively. The partial specific volume *v* was computed as 0.734 ml/g (MagD, MagA:MagD, MagB:MagD, MagF:MagD). Consecutive scans were automatically recorded at regular intervals and analyzed with Sedfit^23^ using the continuous size distribution *c(s)* analysis method to determine *s-* values. Sedfit was also employed to fit a frictional ratio that was combined to the *s-*value in order to generate experimental molar masses, and sedimentation coefficients were corrected to s_*20w*_.

### Small angle X-ray scattering

SAXS data were recorded at the Brazilian Synchrotron Light Laboratory (LNLS) on the SAXS1 beamline equipped with a Pilatus 300 k detector using a wavelength of 1.54 Å and a 903 mm sample-to-detector distance. The s-range extended from 0.1 nm^−1^ to 2.8 nm^−1^. Protein samples were prepared in concentrations of 2 to 6 mg/mL in 25 mM HEPES pH 7.5, 100 mM NaCl, 5% glycerol and 1 mM DTT. Measurements were carried out using a 1.5 mm capillary cell, with a sample holder temperature kept constant at 10 °C. Scattering patterns for protein samples and buffers were collected alternatively with exposure times of 100 s. Data reduction included averaging of individual curves with FIT2D^24^. The radius of gyration, Rg, was estimated using the Guinier approximation *I*(s) = *I*(0) exp(−(s^2^ Rg^2^)/3), valid for small angles (sRg < 1.3), PRIMUS^25^. GNOM^26^ was used to obtain the distance distribution function, *p*(r), and maximum intramolecular distance.

The experimental curve of the MagD data was compared with the calculated scattering curves of the structures of the *S. enterica* and *E. coli* A2M (PDB codes 4U48 and 5A42, respectively) using CRYSOL^22^. In real space, the structures were superimposed with the *ab initio* models of MagD with SUPCOMB^27^.

### Bacterial growth and genetic constructs

Bacterial culture grown in LB medium overnight were diluted to an optical density measured at 600 nm (OD_600_) of 0.15 and incubated further at 37 °C to reach OD_600_ of 1.5. *Pseudomonas aeruginosa* PA01 wild type and Δ*magD* and Δ*operon* mutants were described previously^15^. All DNA fragments to obtain Δ*magA*, Δ*magB*, Δ*magC*, Δ*magE and* Δ*magF* strains were synthesized and cloned in pUC57 by Genscript. In all cases, the ATG and TGA codons were conserved and upstream and downstream flanking regions were between 400 to 500 nucleotides. All sequences harbor *Eco*RI and *Hin*dIII restriction sites at their 5′ and 3′ ends, respectively. Final sizes of the gene to be inactivated were 15 nucleotides (Δ*magA*), 31 nucleotides (Δ*magB*), 18 nucleotides (Δ*magC*), 27 nucleotides (Δ*magE*) and 12 nucleotides (Δ*magF*). Fragments were cloned in pEXG2^28^ using *Eco*RI and *Hind*III restriction sites. pEXG2 vectors were introduced into *P. aeruginosa* PA01 by conjugation using pRK2013 as a helper plasmid and selection on sucrose, as described^29^. For the complementation experiment, a *magB* gene including 19 nucleotides upstream of ATG and the ribosomal binding site and *Eco*RI/*Sac*I restriction sites at the respective 5′ and 3′ ends was synthesized by Genscript. Then *magB* fragment was cloned into a pminiCTX-1 derivative plasmid pSW196 containing the arabinose-inducible promoter pBAD^30^. Plasmids pSW196 harboring *magB* or *magD*^15^ were introduced in *P*. *aeruginosa* by conjugation^29^. When necessary, cultures were grown in the presence of 0.5% arabinose. All bacterial strains, plasmids and primers used in this work are described in Table 4.

**Table 4:** Strains, vectors and oligonucleotides used in this work.

Strains	Characteristics	Origin or Refeence
PA01	Sequenced laboratory strain	J. Mougous, USA
PA01Δ*magA*	Internal deletion in PA4492 (*magA*) in PA01	This work
PA01Δ*magB*	Internal deletion in PA4491 (*magB*) in PA01	This work
PA01Δ*magC*	Internal deletion in PA4490 (*magC)* in PA01	This work
PA01Δ*magD*	Internal deletion in PA4489 (*magD*) in PA01	Robert-Genthon *et al*.^15^
PA01Δ*magE*	Internal deletion in PA4488 (*magE*) in PA01	This work
PA01Δ*magF*	Internal deletion in PA4487 (*magF*) in PA01	This work
PA01 pEX100T_ΔOp	Internal deletion of whole operon mag PA4492-PA4487	Robert-Genthon *et al*.^15^
*E. coli* Top10	Cloning strain	Invitrogen
*E. coli* pRK2013	*E. coli* with plasmid Helper for conjugaison	Addgene
PLASMIDS		
pUC57	Cloning vector, Amp^R^	Genescript
pCCI	Cloning vector, Cm^R^	Genecsript
pEXG2	Plasmid for allelic exchange, *sac*B, Gm^R^	Rietsch *et al*.^28^
pEXG2-Δ*magA*	Plasmid containing *magA* deletion	This work
pEXG2-Δ*magB*	Plasmid containing *magB* deletion	This work
pEXG2-Δ*magC*	Plasmid containing *magC* deletion	This work
pEXG2-Δ*magE*	Plasmid containing *magE* deletion	This work
pEXG2-Δ*magF*	Plasmid containing *magF* deletion	This work
pEX100T	Mini-CTX1 based plasmid for allellic exchange, pBAD, Tc^R^	Baynham *et al*.^30^
pSW196-*magB*.RBS	Plasmid for *magB*.RBS (PA4491) gene complementation	This study
pSW196-*magD*	Plasmid for *magD* (PA4489) gene complementation	Robert-Genthon *et al*.^15^
PRIMERS	SEQUENCE 5′-3′	CHARACTERISTIC
F. D*magA*	5′ GTGGATTTCGCACATTCCGCC	Verification *magA* deletion
R. D*magA*	5′ ACCTCGAGGATTTCCTTCGGC	Verification *magA* deletion
F. D*magB*	5′ ACAGGACGTGATCACCGCGC	Verification *magB* deletion
R. D*magB*	5′ CGATGCGCACGAACCAGGC	Verification *magB* deletion
F. D*magC*	5′ GACACCCTCGACAAACGC	Verification *magC* deletion
R. D*magC*	5′ GCTTGAGGAACTCCAGCG	Verification *magC* deletion
F. D*magE*	5′ GCGTTCGAGTTCAAGGTCG	Verification *magE* deletion
R. D*magE*	5′ CCACCTTCAGTGGCATGC	Verification *magE* deletion
F. D*magF*	5′ CTGTATTCCCTACAGGAACGCC	Verification *magF* deletion
R. D*magF*	5′ CGGTGCGCTGAACAACGGC	Verification *magF* deletion

### **Fractionation of*Pseudomonas aeruginosa***

For analysis of total bacterial extracts, overnight cultures were diluted to an OD_600nm_ of 0.15 AU and cultivated at 37 °C, 300 rpm until they reached an OD_600nm_ of 1.5 AU. Cultures were concentrated 10-fold by centrifugation, resuspended directly in SDS-PAGE loading buffer (62 mM Tris-HCl pH 6.8, 2% SDS, 5% ß-mercaptoethanol) and incubated 10 min at 100 °C. Fractionation of bacterial cells was performed using exponential grown cultures (OD_600_ = 1). The pellet equivalent to 20.10^9^ bacteria was resuspended in 1 ml Buffer M (20 mM Tris-HCl pH 8.0, 200 mM MgCl_2_ containing Protease Inhibitor Cocktail - PIC, Roche) with 0.5 mg/ml lysozyme and incubated 15 min at 4 °C, under gentle rotation. The periplasmic fraction was obtained after centrifugation at 8,000 g for 15 min at 4 °C. After one wash with buffer A the pellet was resuspended in buffer A (20 mM Tris-HCl pH 8.0, 20 mM MgCl_2_, PIC Roche) and disrupted by sonication (VibraCell 75185, 5 min, 5 sec on/5 sec off, 40% power). Unbroken bacteria were eliminated by centrifugation at 8000 g for 15 min. Then supernatant was ultracentrifuged at 200,000 g, 45 min at 4 °C to obtain the cytosolic fraction in the supernatant and the total membrane fraction in the pellet. All samples were diluted in SDS-PAGE denaturing buffer and heated 10 min at 100 °C before loading onto SDS-PAGE.

### Immunoblot analyses

Proteins were separated on SDS-PAGE (Criterion 8–16% TGX precast gel Biorad or 10% polyacrylamide gel) and transferred onto a PVDF membrane (Hybond LFP-Polyvinylidene Difluoride, GE Healthcare) by 0.3 A electrotransfer in 20% ethanol Laemmli buffer (20 mM Tris pH 8.3, 192 mM glycine, 0.1% SDS). Membranes were blocked with 5% nonfat dry milk in PBS buffer containing 0.1% Tween 20. Dilutions of primary polyclonal antibodies were as follows: anti-MagD^15^ 1:40,000, anti-EF-Tu (Hycult Biotech HM6010) 1:20,000, anti-DsbA (a kind gift from R. Voulhoux, Marseille, France) 1:2,000 and anti-TagQ^31^ 1:10,000 in PBS-Tween buffer. Incubations were performed overnight at 4 °C. Secondary antibodies (anti rabbit-HRP, anti mouse-HRP, Sigma) were incubated for 1 hour at room temperature. Detection was performed using the Luminata Classico HRP-Substrate (Millipore).

### Mass spectrometry and N-terminal sequencing

Experiments were performed using the platforms of the Partnership for Structural Biology. For N-terminal sequencing, samples were transferred from a SDS-PAGE gel to a PVDF membrane. After staining, bands of interest were excised and subsequently injected into a Procise 492 sequencer (Applied Biosystems), and analyzed by Edman degradation. Protein samples for mass spectrometry were prepared in 25 mM HEPES pH 7.5, 300 mM NaCl. 8 μL of samples a concentration of 10 uM were injected into an ESI Q-TOF mass spectrometer from Waters (Ultima).

## Electronic supplementary material

Supplementary information
