# Supplementary material for: Assembly of an atypical α-macroglobulin complex from Pseudomonas aeruginosa
Source: Sci Rep. 2018 Jan 11;8:527. doi: 10.1038/s41598-017-18083-6 (PMC5764988; doi:10.1038/s41598-017-18083-6)
Supplement: Supplementary file 1 — Supplementary information [file 41598_2017_18083_MOESM1_ESM.pdf]

Supplementary Data for

Assembly of an atypical α-macroglobulin complex from *Pseudomonas aeruginosa*

Samira Zouhir, Mylène Robert-Genthon, Daniel Maragno Trindade, Viviana Job, Marko Nedeljković, Cécile Breyton, Christine Ebel, Ina Attrée, and Andréa Dessen

Figure S1

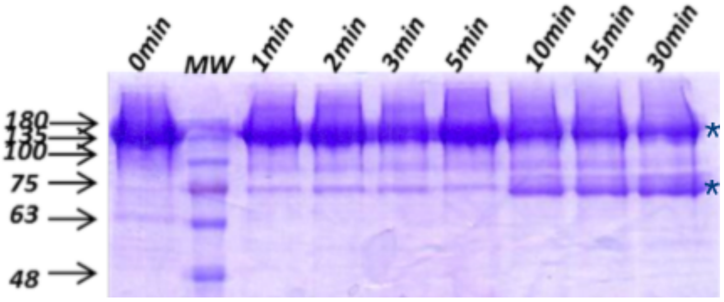

Supplementary Fig. 1: Limited proteolysis results for MagD. MagD was incubated with chymotrypsin, trypsin, and papain in a 1:200 (m/m) ratio, and proteolysis was verified at different time points in 12.5% SDS-PAGE gels. For t0, proteolysis was inhibited by PMSF prior to incubation with MagD. In all three cases, N-terminal sequencing detected three distinct species: GVKVLERP, corresponding to the C-terminus of MagD starting at 843 (and thus cleaved at the bait region), as well as DAQPQYAP and LAGSSSEF, which corresponded to the N-terminal region starting at residues 164 and 199. This attests to the flexibility of the region upstream from residue 199 in the MagD sequence. For clarity, only the results for trypsin are shown. Full-length and bait-region cleaved forms are indicated with a star (*).

Figure S2

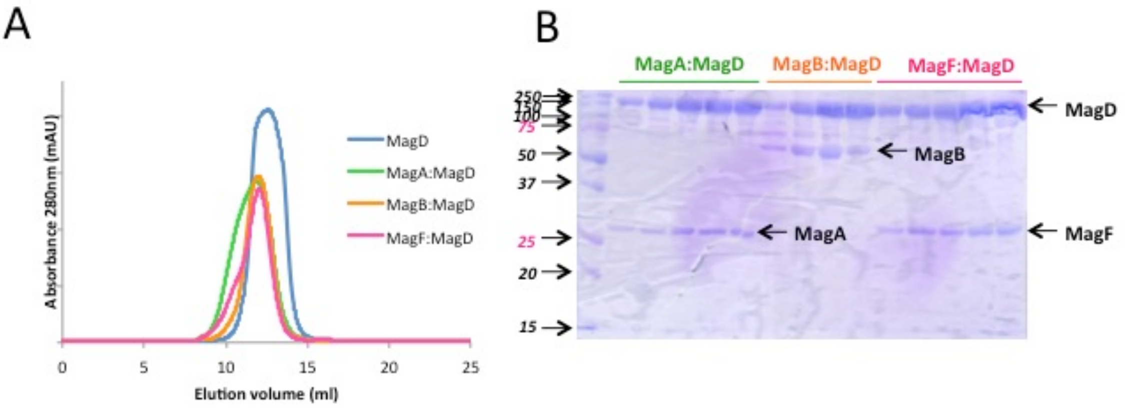

Supplementary Figure 2: Analytical size exclusion chromatography profiles and SDS-gels of MagD co-expressed with MagA, MagB, or MagF. The profile is presented as the elution volume (ml) as a function of the absorbance at 280 nm (mAU). (A) Experiments were performed in a Superdex S200 10/300 column (GE Healthcare). (B) SDS-PAGE analysis of the elution fractions indicates co-elution of MagD with its partners. MagD = 165 kDa; MagA = 27 kDa; MagB = 60 kDa and MagF = 26 kDa.

Figure S3

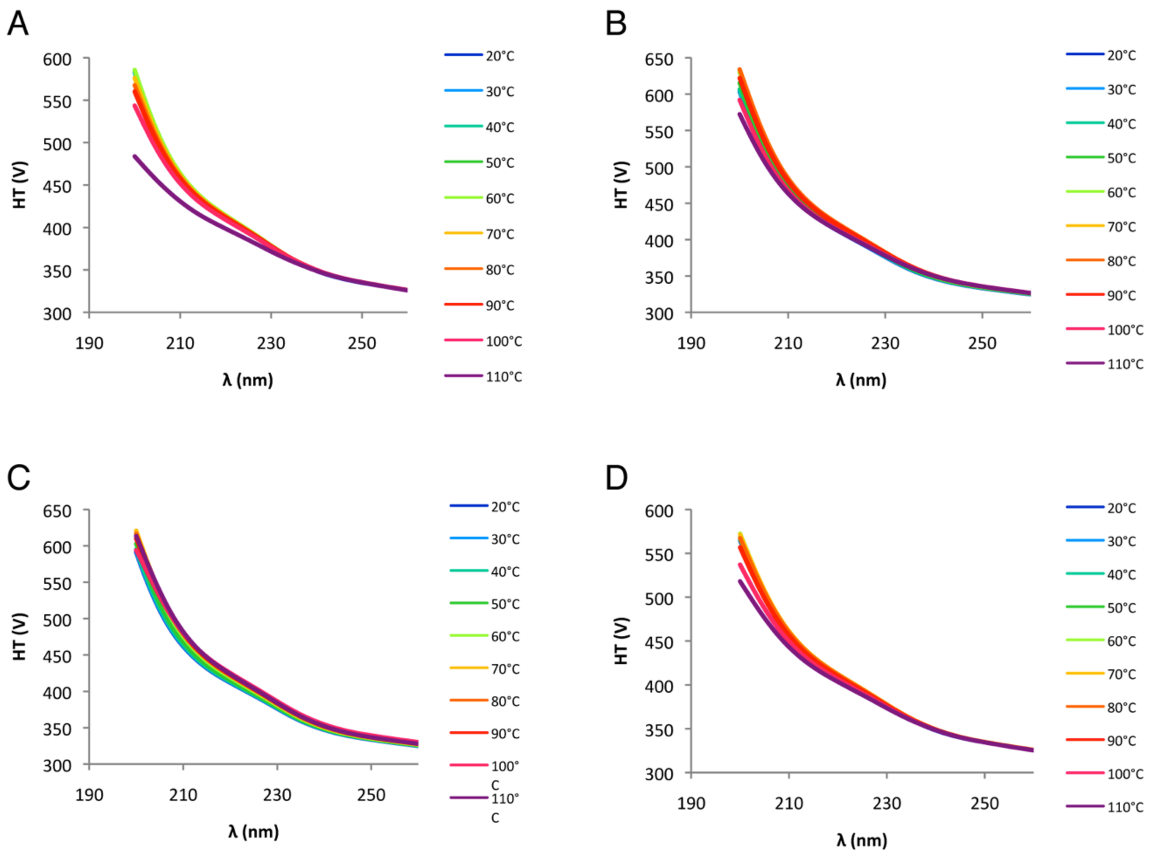

Supplementary Figure 3: High tension variation plots of CD thermal unfolding spectra: MagD (A), MagA:MagD (B), MagB:MagD (C), MagF:MagD (D). High tension (V) plotted in function of the wavelength (nm) is displayed for CD spectra from 20°C to 110°C (10°C steps). Increases in high tension, that could indicate aggregation of the samples, were not observed.
